# Alternative health care consultations in Ontario, Canada: A geographic and socio-demographic analysis

**DOI:** 10.1186/1472-6882-11-47

**Published:** 2011-06-22

**Authors:** Allison M Williams, Peter Kitchen, Jeanette Eby

**Affiliations:** 1 Department of Geography and Earth Sciences, McMaster University, Hamilton, Ontario, Canada

## Abstract

**Background:**

An important but understudied component of Canada's health system is alternative care. The objective of this paper is to examine the geographic and socio-demographic characteristics of alternative care consultation in Ontario, Canada's largest province.

**Methods:**

Data is drawn from the Canadian Community Health Survey (CCHS Cycle 3.1, 2005) for people aged 18 or over (n = 32,598) who had a consultation with an alternative health care provider. Four groups of consultations are examined: (1) all consultations (2) massage therapy (3) acupuncture, and (4) homeopath/naturopath. Descriptive statistics, mapping and logistic regression modeling are employed to analyze the data and to compare modalities of alternative health care use.

**Results:**

In 2005, more than 1.2 million adults aged 18 or over consulted an alternative health care provider, representing about 13% of the total population of Ontario. The analysis revealed a varied geographic pattern of consultations across the province. Consultations were fairly even across the urban to rural continuum and rural residents were just as likely to consult a provider as their urban counterparts. From a health perspective, people with a chronic condition, lower health status and self-perceived unmet health care needs were more likely to see an alternative health provider. Women with chronic conditions such as fibromyalgia, high blood pressure, chronic fatigue syndrome and chemical sensitivities were more likely to see an alternative provider if they felt their health care needs were not being met.

**Conclusions:**

The analysis revealed that geography is not a factor in determining alternative health care consultations in Ontario. By contrast, there is a strong association between these consultations and socio-demographic characteristics particularly age, sex, education, health and self-perceived unmet health care needs. The results underscore the importance of women's health needs as related to alternative care use. The paper concludes that there is a need for more place-specific research that explores the reasons why people use specific types of alternative health care as tied to socio-economic status, health, place of residence, and knowledge of these treatments.

## Background

In Canada and other developed countries, the use of alternative health care is on the rise. Alternative health care, also commonly known as complementary and alternative medicine (CAM), encompasses a variety of health care practices that are not within the conventional biomedical realm. A defining feature of alternative care is that it focuses on 'whole person health' [1]. While extensive research has been conducted over the past few decades on the practice of alternative health care, less work has been carried out on the geographic and socio-demographic characteristics of users of alternative care. This paper employs data from the 2005 Canadian Community Health Survey (CCHS) to examine alternative health care in Ontario. It contributes to the socio-geographic perspective that is currently lacking in many studies by analyzing data specific to the use of three types alternative health care: (1) massage therapy, (2) acupuncture and (3) homeopathy/naturopathy. Definitions for these alternative therapies are provided by their respective associations in Canada [2-5]. The geographic analysis employs two variables: Census rural and metropolitan influence zone. The data analysis involves four steps: 1) descriptive statistics, 2) mapping, 3) logistic regression and 4) contingency tables.

## Review of Literature

Some common trends are evident in the limited literature that has drawn attention to the socio-demographic and health characteristics of alternative health care users. Canadian and international studies have demonstrated similar findings with respect to utilization. These include the fact that users are more likely to be middle-aged women [6-8], and people with higher levels of education and higher incomes [6-16]. North American research also shows that individuals living with a chronic illness, those with poor self-rated health and people who have self-perceived unmet health care needs are more likely to consult a practitioner of alternative medicine [8,10-12,15,16]. However, several studies have challenged the commonly held notions of alternative care use. For example, Wolsko et al's research addressed alternative health care use among poor and underserved populations. Through a medical clinic survey, the authors found that there is a wide use of alternative health care outside of the typical higher socio-economic demographic [17]. Fox et al's study on alternative health care in Ireland revealed that use is more prevalent among self-employed individuals, a finding distinct from other studies [16]. Andrews and Boon also note that alternative health care use is broadening beyond the high-earning and highly-educated demographic, perhaps due to better education about alternative health care, combined with increased accessibility in different geographic locations [18].

Jones et al's study of alternative health care in the United States focused on utilization by people with chronic fatiguing illnesses. Their study emphasized the prevalence of women using these health care options, specifically those with low physical and mental health scores [10]. Similarly, Wu et al revealed that there is a high rate of alternative health care use among American women suffering from depression [19]. A more recent Canadian-based survey focused on individuals with chronic diseases (diabetes, epilepsy, migraines and asthma) and found that alternative health care use varies greatly depending on the disease. The study revealed that individuals with asthma and migraines have significantly higher rates of alternative health care use than the general population and that people with diabetes have a lower rate [11]. The use of alternative health care is also increasing for specific patient groups, especially for those who have been diagnosed with different types of cancer [18]. Interestingly, there have been few comparative studies that explore the characteristics of people who use both alternative and mainstream health care. A Scandinavian study compared three groups of individuals: 1) people who use only alternative health care, 2) individuals who use only general practitioners, and 3) those who use a combination of both. The study found that people who use both types of heath care share the common user characteristics evident in other studies. It also demonstrated that there is an association between alternative health care use and hay fever and psychiatric needs [20].

Bodeker et al highlight the fact that there is a general lack of data addressing levels of access and affordability of alternative health care and that many of these services are paid for out-of-pocket [21]. In Canada, there is a significant cost associated with most types of alternative health care, as it is generally not covered by provincial health insurance [9]. While some provinces cover chiropractor services, most users resort to private insurance or employee health benefits to pay for massage therapy, homeopathy, naturopathy and acupuncture [8]. Very little research has been devoted to the relationship between the use of alternative health care and insurance coverage. An exception is Xue et al's Australian study, which found that use of alternative health care is more prevalent among individuals with private health insurance [14].

In the wider literature, socio-demographic analysis of alternative health care users is more prevalent than studies with a geographic focus. A 2004 South Australian Omnibus survey revealed that a higher percentage of users lived in rural rather than metropolitan areas. Relatively little research has focused on rural settings or variations between urban and rural areas. It is important to understand these variations in order to address issues in health services management and geographic barriers to health care services [22]. In their review of North American and Australian studies, Wardle et al described the similar characteristics of alternative health care users in rural areas compared to those of the general population. The authors also referred to the prevalence of use among older adults in rural areas [22].

Wiles and Rosenberg's place-based exploration of alternative health care in Canada demonstrated that the availability of these services is more prominent in big cities compared to smaller urban places and rural communities [9]. A number of studies in Canada have found that the use of alternative health care is more prevalent in the Western provinces [8,23]. Meyer's spatial analysis of alternative health care offices in Ontario revealed that most practices are located in certain sections of metropolitan areas, specifically in areas around the central city where there is a high density of people, high visibility and high traffic flow [23]. Meyer addressed the issue of inequality in health care provision across space and compared the availability of conventional medicine to alternative health care in Ontario [24]. He found that the offices of alternative health care providers are more evenly distributed geographically compared to the offices of conventional medical practitioners. Meyer also examined the issue of health care consumption and questioned practitioners' motives behind opening their practice in certain metropolitan centres, as many alternative health care locations are in economically vibrant regions [24]. Geographers have highlighted the link between alternative health care and health consumerism and the fact that while practitioners are health professionals, they also must be proficient small business operators in order to sustain their work [9,23-25].

Regulatory policies for alternative health care, involving registration, certification and licensing differ across Canada. Naturopathy is regulated in British Colombia, Saskatchewan, Manitoba, Ontario and Nova Scotia; acupuncture is regulated only in British Colombia, Alberta, and Quebec, and massage therapy is regulated in Ontario, Newfoundland/Labrador and British Colombia [26,27]. As alternative health care use increases, the diversity of practices, places and individuals who access these services is also increasing and thus there is a demand for more in-depth research. We now turn to the Canadian Community Health Survey (CCHS) to explore alternative health care use in Ontario from a socio-geographic perspective.

## Methods

The data for this research are drawn from the 2005 Canadian Community Health Survey (CCHS) Master file. The CCHS is an annual cross-sectional survey of Canadians aged 12 or over in all provinces and territories. Its primary objective is to gather health-related data on a wide range of topics and issues. The CCHS is made available to researchers in two forms: a Public Use Micro Data File and a master file. The master file contains micro data that is not available in the public use file, including several geography related variables. The data in the master file is not openly available to researchers, although it can be accessed following an application process. The authors were granted permission to use this data through Statistics Canada's Research Data Centres Program, a process adjudicated by the Social Sciences and Humanities Research Council. A formal ethics application is not required. The CCHS is a large survey where all data are self-reported and the sample is representative of the Canadian household population. The 2005 CCHS included a module where respondents were asked questions about alternative health care consultations that they had made in the past 12 months. Table 1 lists the CCHS variables that are analyzed for this research. They comprise the dependent variable (consultation with an alternative health provider) and 10 independent variables measuring, socio-economic status, health and geography. As stated, three types of alternative health care are examined: massage therapy, acupuncture and homeopathy/naturopathy. Other types of alternative care were listed in the CCHS including: relaxation therapy, biofeedback, reflexology and herbalist. However, these could not be included in the data analysis due to small sample sizes that did not adhere to Statistics Canada's data disclosure policy. It is interesting to note that chiropractics are not included as a form of alternative health care in the CCHS, likely the result of this type of care now being recognized as mainstream rather than 'alternative' or 'complementary'.

**Table 1:** Canadian Community Health Survey (CCHS)

**Dependent Variable**		
**Variable name Concept**	**Survey Question**	**If 'Yes', who did you see or talk to?**
HCUC_04 Consulted alternative health care provider	In the past 12 months, have you seen or talked to an alternative health care provider such as an acupuncturist, homeopath or massage therapist about your physical, emotional or mental health? ('Yes', 'No', 'N/A')	1) Massage therapist 2) Acupuncturist 3) Homeopath or naturopath
**Independent Variables**		
**Variable**	**Survey Question**	**Coded Responses**
Sex	Is respondent male or female?	Male -- Female
Age	What is your age?	Age18 to 24 - Age 25 to 44 - Age 45 to 64 - Age 65 and over
Marital status	What is your marital status?	Single - Married/common Law -- Separated/divorced/widowed
Education	Derived variable	Less than high school -- High school -- Other post-secondary -- College or university
Household income	What is your best estimate of the total income, before taxes and deductions, of all household members from all sources in the past 12 months?	Less than $20,000--$20,000 to $49,000--$50,000 to $79,000 -- More than $80,000
Chronic condition	Derived variable	Yes -- No
Self-perceived health	In general, would you say your health is?	Excellent/Very good -- Good -- Fair/poor
Unmet health care needs	During the past 12 months, was there ever a time when you felt that you needed health care but you didn't receive it?	Yes - No
Census rural	Derived variable	Urban -- Rural
Metropolitan Influence Zone (MIZ)	Derived variable Statistical area classification type	CMA/CA -- strongly influenced -- Moderate influence -- Weak or no influence

This study examines the total population aged 18 or over who consulted an alternative health care provider. Data from CCHS Cycle 2.1 (2005, n = 37,855) were analyzed representing respondents who reported consulting an alternative care provider (n = 4,771) and those who did not (n = 33,084). The CCHS master weight for each individual respondent was used to produce frequency tables comprising estimates of the total population aged 18 or over in Ontario who consulted an alternative health care provider. For all other analysis, a normalized weight was employed. This was achieved by dividing the master weight for each survey respondent by the mean weight of the sample population (those aged 18 or over in Ontario).

As stated, one of the objectives is to assess the geographic dimension of alternative health care consultations in Ontario. This is achieved in two ways. The first is by mapping alternative health care use among the province's 36 Public Health Units (PHUs) to examine broad regional variations. The second is by incorporating two measures of rurality into the analysis: Census rural and Metropolitan Influence Zone (MIZ). In the CCHS, Census rural is a dichotomized variable in which an urban area is defined as a continuously built-up area having a population concentration of 1,000 or more and a population density of 400 or more per square kilometre. As a result, a 'rural' person is someone who does not live in an urban area according to that definition. MIZ refers to the population living outside the commuting zone of a larger urban center, such as a census metropolitan area or census agglomeration. A census metropolitan area must have a total population of 100,000 and a census agglomeration must have an urban core population of at least 10,000. Statistics Canada classifies four zones: Strong MIZ, Moderate MIZ, Weak MIZ and No MIZ. Statistics Canada classifies the four zones as follows: in a Strong MIZ, at least 30% of the municipality's resident employed labour force commute to work in any CMA or CA; in a Moderate MIZ, at least 5%, but less than 30% of the municipality's resident employed labour force commute to work in any CMA or CA; in a Weak MIZ, more than 0%, but less than 5% of the municipality's resident employed labour force commute to work in any CMA or CA, and; for No MIZ, fewer than 40 or none of the municipality's resident employed labour force commute to work in any CMA or CA.

In this measure, a 'rural' person is someone who does not live in a census metropolitan area or census agglomeration. In other words, they reside in a town or municipality with a population under 10,000.

The data analysis involved four steps: 1) descriptive statistics, 2) mapping 3) logistic regression and 4) contingency tables. In the first step, descriptive statistics were produced showing the basic socio-demographic and geographic characteristics of alternative health care consultations in Ontario. In the second step, mapping software (ESRI ArcGIS) was employed to create a choropleth map showing the geographic distribution of these consultations in the province's 36 Public Health Unit (PHUs). The third step involved logistic regression analyses of the CCHS data. Four models were devised where the dependent variable represents people who consulted an alternative health care provider and the independent variables denote a number of socio-demographic (gender, age, marital status, education, income), health (presence of a chronic condition, self-rated health, self-perceived unmet health care needs) and geographic (Census rural, MIZ) characteristics. Regression coefficients are employed to estimate odds ratios for each of the independent variables in the model. The objective is to identify the factors associated with the use of alternative health care in Ontario, be they socio-economic, health or geographic. The first regression (Model 1) compares all users of alternative care to all non-users in Ontario. Models 2, 3 and 4 compare the users of each individual type of alternative care (massage, acupuncture, homeopath/naturopath) to all other users of alternative care. The fourth step in the analysis consists of contingency tables, which examine the association between self-perceived unmet health care needs and the use of alternative health care among women in Ontario who suffer from certain chronic health conditions. Chi-square analysis is used to test the statistical significance of these relationships.

## Results

### Overall Use

Table 2 provides a summary of alternative health care consultations in Ontario. In 2005, more than 1.2 million people aged 18 or over consulted an alternative health care provider, representing about 13% of the total population in this age group. Nearly two-thirds (65%) of consultations were by women and 41% were by people aged 35 to 49. In 2005, about 86% of consultations were by urban residents. This rate is higher than the urban-rural population distribution in Ontario (82% urban and 18% rural). Table 2 also summarizes consultations with three types of alternative care providers. The most frequent, by a large margin, was with a massage therapist. Nearly 820,000 people in Ontario consulted this type of provider with the sex, age and location characteristics very similar to those of all consultations. Just over 248,000 people consulted with a homeopath or naturopath provider in the province. Women (69.4%), those aged 35 to 49 (44.5%) and rural residents (17.5%) had more consultations with this type of provider compared to other forms of alternative care. Table 2 shows somewhat different characteristics with respect to contact with an acupuncturist with males (43%), people over 50 (39%) and urban residents (87.5%) having more consultations compared to massage therapy and homeopathy/naturopathy.

**Table 2:** Consulted an Alternative Health Care Provider: Population Aged 18 or over in Ontario, 2005

	All Consultations		Massage Therapist		Acupuncturist		Homeopath/Naturopath	
	Total	Percent	Total	Percent	Total	Percent	Total	Percent
Total	1,237,771	12.9*	819,489	8.5*	219,179	2.3*	248,036	2.6*
Sex								
Male	437,487	35.3**	271,686	33.2**	94,069	42.9**	75,965	30.6**
Female	800,284	64.7**	547,803	66.8**	125,110	57.1**	172,071	69.4**
Age								
18-34	374,672	30.3**	273,732	33.4**	45,314	20.7**	68,616	27.7**
35-49	509,998	41.2**	344,485	42.1**	87,480	39.9**	110,327	44.5**
50-64	265,224	21.4**	164,967	20.1**	60,296	27.5**	51,963	20.9**
65 and over	87,877	7.1**	36,306	4.4**	26,090	11.9**	17,129	6.9**
Residence								
Urban	1,062,617	85.8**	706,467	86.2**	191,792	87.5**	204,563	82.5**
Rural	175,154	14.2**	113,022	13.8**	27,387	12.5**	43,474	17.5**

* Percent of total population in Ontario aged 18 or over.

** Percent of alternative care consultations

### Geography

**Figure 1:**
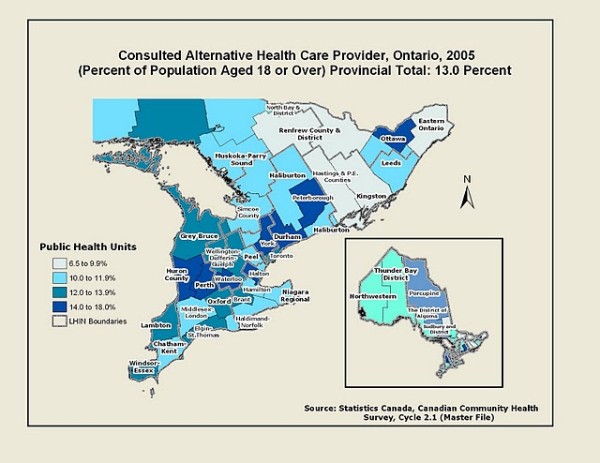
**Map of alternative health care provider consultations in Ontario, 2005**.

The analysis of the CCHS data reveals a variable geographic pattern of alternative health care consultations. Figure 1 utilizes Ontario's Public Health Units to demonstrate spatial variations across the province. It is important to point out that Ontario's 36 Public Health Units are not involved in the organization, management or delivery of alternative care, which is largely self-governed. They are used here to convey broad geographic patterns of alternative care use in the province. Figure 1 shows a clustering of PHUs in central, southern and southwestern Ontario; all of these PHUs had the highest proportion of alternative care consultations across the province (all above 15% of the total population 18 or over in each PHU). Three of these are within the heavily populated Greater Toronto Area (GTA): Halton (16.4%), York (15.7%) and Durham (15.4%). The lowest rates of alternative health care consultations were apparent in the large, rural and remote PHUs in the eastern and northern parts of the province. These include, in the east: Eastern Ontario (9.3%), Renfrew (8.7%), Kingston, Frontenac and Lennox & Addington (7.7%) and Hastings and Prince Edward Counties (6.8%), and, in the north: Northwestern (10.4%) and Timiskaming (6.5%).

### Alternative Health Care across the Urban to Rural Continuum

Tables 3 and 4 compare the use of alternative health care across the urban to rural continuum in Ontario using the four metropolitan influence zones. Table 3 reveals a fairly even distribution of all types of consultations across the four zones in terms of the proportion of each area's total population having a consultation (between 11.3% and 13.3%). In Table 4, the sample is reduced to just people who had an alternative care consultation and omits all those who did not. As the most popular type of alternative health care, massage therapy consultations were highest in CMA/CAs (66.6%) and Strong MIZ (68.1%) and considerably lower in the Moderate MIZ (58.7%). Interestingly, consultations with a massage therapist were relatively high in the Weak or No MIZ at 63.3% of all alternative health care users in that zone. This result suggests that unlike other health care services, massage therapy is available in the more remote regions of the province, although long travel distances may be a factor for many users. Table 4 reveals a fairly wide gap in consultations with an acupuncturist across the urban to rural continuum with rates highest in CMA/CAs (18.3%) compared to just 10.1% in the Weak or No MIZ. With respect to consultations with a homeopath or naturopath provider, the data in Table 4 demonstrate a trend opposite to the other two types of care; there is a progressive growth in the use of these services across the urban to rural continuum from 19.6% in CMAs/CAs to 26.6% in the Weak or No MIZ.

**Table 3:** Alternative Health Care Consultations Across the Urban to Rural Continuum in Ontario, 2005

Metro. Inf. Zone	Total Residents Age 18 or over	All Alt. Care Consultations	Proportion
CMA/CA	8,438,341	1,099,095	13.0%
Strongly Inf.	519,023	68,978	13.3%
Moderately Inf.	372,949	42,188	11.3%
Weak or No Inf.	232,903	27,510	11.8%
Total	9,563,216	1,237,771	12.9%

**Table 4:** Alternative Health Care Consultations Across the Urban to Rural Continuum in Ontario by Type, 2005 (Total and percent of all consultations in each MIZ)

Metro. Inf. Zone	Massage Therapist	Acupuncturist	Homeopath/Naturopath
CMA/CA	730,181 (66.6%)	200,576 (18.2%)	214,556 (19.5%)
Strongly Inf.	47,081 (68.2%)	9,289 (13.5%)	15,056 (21.8%)
Moderately Inf.	24,753 (58.7%)	6,607 (15.7%)	11,141(26.4%)
Weak or No Inf.	17,474 (63.5%)	2,707 (9.8%)	7,283 (26.5%)
Total	819,489 (66.2%)	219,179 (17.7%)	248,036 (20.3%)

### Determinants of Alternative Health Care Consultations

Logistic regression analysis was performed on the 2005 CCHS data to assess the determinants of alternative health care consultation in Ontario. Four models were produced which tested the association between 10 independent variables and the likelihood of having an alternative health care consultation with any type of provider (Model 1), a massage therapist (Model 2), an acupuncturist (Model 3) and a homeopath or naturopath provider (Model 4). The sample characteristics of the 10 independent variables are listed in Table 5. They comprise five socio-economic measures (gender, age, marital status, education and household income), three health-related indicators (presence of a chronic condition, self-rated health and self-perceived unmet health care needs) and two geographic variables (Census rural and MIZ).

**Table 5:** Sample Characteristics for Independent Variables: Population aged 18 or over in Ontario (all respondents, n = 32,598)

Variable		Variable	
**Sex**		**Has a Chronic Condition**	
Male	14,999 (46.0%)	Yes	25,058 (76.9%)
Female	17,599 (54.0%)	No	7,540 (23.1%)
**Age**		**Self-Perceived Health**	
18-34	8,399 (25.8%)	Excellent/very good	18,968 (58.2%)
35-49	8,948 (27.4%)	Good	9,041 (27.7%)
50-64	8,099 (24.8%)	Fair/poor	4,589 (14.1%)
65 and over	7,152 (21.9%)	**Unmet health care needs**	
**Marital Status**		Yes	4,032 (12.4%)
Single	7,013 (21.5%)	No	28,566 (87.6%)
Married/common Law	18,818 (57.7%)	**Rural Residence**	
Separated/divorced/widowed	6,767 (20.8%)	Urban	26,213 (80.4%)
**Education**		Rural	6,385 (19.6%)
Less than high school	5,851 (17.9%)	**Place of Residence**	
High school	5,862 (18.0%)	CMA/CA	25,148 (77.1%)
College or trades	13,671 (41.9%)	Strongly Influenced MIZ	2,827 (8.7%)
University	7,214 (22.1%)	Moderately Influenced MIZ	2,731 (8.4%)
**Household Income**		Weakly/Not Influenced MIZ	1,892 (5.8%)
Under $20,000	4,230 (13.0%)		
$20,000 to $49,999	10,440 (32.0%)		
$50,000 to $79,999	8,277 (25.4%)		
$80,000 and over	9,651 (29.6%)		

Table 6 shows the results of Model 1 where respondents who had any type of an alternative health care consultation are coded as 1 and all respondents who did not have a consultation are coded as 0 in the dependent variable. Thus, the sample for Model 1 includes all respondents in Ontario aged 18 or over. Independent variables having significant odds ratios (p < 0.10, p < 0.05 and p < 0.01) are marked with asterisks. The results indicate that women were twice as likely (OR = 2.03) to have an alternative care consultation than men (the reference group) and that younger (20 to 34 years old, OR = 2.33), middle aged (35-49 years old, OR = 2.60) and older adults (50-64 years old, OR = 1.89) were more likely to have a consultation than those age 65 or over (the reference group). Interestingly, respondents who were separated/divorced/widowed were 1.2 times more likely (OR = 1.22) to have a consultation than single people. Model 1 reveals that education and income were both significantly associated with alternative health care use. People with a college or trades certificate were more than twice as likely (OR = 2.38) and those with a university education were more than three times as likely (OR = 3.48) to consult a practitioner than people with less than a high school education (the reference group). At the same time, there was a direct and positive association between increasing household income and the likelihood of seeing an alternative health care provider. People living in a household with an income of $80,000 or more were more than twice as likely (OR = 2.30) to have such as a consultation than those residing in a household with an income less than $20,000.

**Table 6:** Results of Logistic Regression Analyses Alternative Health Care Consultations: All Consultations Age 18 or over, Ontario, 2005^a^

	Model 1 All Alt. Care Consultations (consultations = 1 no alt. care consultations = 0)
Independent Variables	Odds ratios
**Sex**	
Male	Reference
Female	2.03*** (1.90-2.18)
**Age**	
Age 20-34	2.33*** (1.99-2.73)
Age 35-49	2.60*** (2.25-3.01)
Age 50-64	1.89*** (1.63-2.20)
Age 65 and over	Reference
**Marital status**	
Single	Reference
Married/common Law	1.03 (0.94-1.13)
Separated/divorced/widowed	1.22** (1.06-1.40)
**Education**	
Less than high school	Reference
High school	1.49*** (1.26-1.76)
College or trades	2.38*** (2.05-2.76)
University	3.48*** (2.99-4.06)
**Household income**	
Under $20,000	Reference
$20,000 to $49,999	1.23** (1.04-1.46)
$50,000 to $79,999	1.82*** (1.53-2.15)
$80,000 and over	2.30*** (1.95-2.72)
**Chronic condition**	
Yes	1.90*** (1.74-2.07)
No	Reference
**Self-perceived health**	
Excellent/very good	Reference
Good	0.06 (0.98-1.15)
Fair/poor	1.27*** (1.13-1.43)
	
**Self-perceived unmet health care needs**	
Yes	1.72*** (1.58-1.88)
No	Reference
**Rural residence**	
Urban	Reference
Rural	1.06 (0.95-1.18)
**Place of residence**	
CMA/CA	Reference
Strongly Influenced	1.07 (0.91-1.25)
Moderately Influenced	1.05 (0.87-1.27)
Weakly/Not Influenced	1.03 (0.82-1.29)
	
Constant	0.006
Observations	32,598
Pseudo R2	0.119

^a ^Notes: The dependent variable is consulting an alternative health care provider. The model used for estimation is logistic regression. 95% confidence intervals in parentheses (*) significant at 10%, (**) significant at 5%, (***) significant at 1%. Reference categories are included in the table.

Each of the three health-related measures had a positive influence on alternative care consultation. People with a chronic condition were almost twice as likely (OR = 1.90) to seek alternative care than those having no chronic condition while people with 'fair/poor' health were more likely (OR = 1.27) to a see a provider than those reporting 'excellent/very good' health. As Model 1 shows, perceptions of unmet health care needs play an important role in the use of alternative care. People who said they had 'unmet needs' were 1.7 times more likely (OR = 1.72) to have a consultation than those who said their health needs were being met. Finally, Model 1 indicates that geography was not a factor in alternative care consultations in Ontario with neither of the two variables (Census rural or MIZ) having significant odds ratios.

Table 7 shows the results of regression Models 2 to 4. In these, the sample size is reduced to only those who reported having an alternative care consultation. (Model 1 included people who reported a consultation and those who did not). In Model 2, the dependent variable signifies respondents who consulted with a massage therapist (code 1) and those who had any other type of alternative health care consultation (code 0). From a socio-demographic perspective, the results indicate than women (OR = 1.43), those under the age of 50 (age 20 to 34, OR = 2.48: age 35 to 49, OR = 1.94) and people with higher household incomes ($80,000 or more, OR = 2.65) were more likely to see a massage therapist than another type of alternative provider. Marital status and education did not have a significant influence. Model 2 shows that the three health-related measures are reversed for massage therapy compared to overall consultations (Model 1). The presence of a chronic condition was not associated with massage therapy. Furthermore, those reporting 'fair/poor' health (OR = 0.55) and those saying they had unmet health care needs (OR = 0.79) were less likely to consult a massage therapist. These results suggest that people with unmet health needs or with poorer health seek other modalities of alternative care. Like Model 1, geography did not have an influence on massage therapy consultations.

**Table 7:** Results of Logistic Regression Analyses Alternative Health Care Consultations: Massage Therapist, Acupuncturist and Homeopath/Naturopath Age 18 or over, Ontario, 2005^a^

	Model 2 Massage Therapist (massage = 1 all other alt. care = 0)	Model 3 Acupuncturist (acupuncture = 1 all other alt. care = 0)	Model 4 Homeopath/Naturopath (homeopath/naturopath = 1 all other alt. care = 0)
Independent Variables	Odds ratios	Odds ratios	Odds ratios
**Sex**			
Male	Reference	Reference	Reference
Female	1.43*** (1.25-1.64)	0.60*** (0.51-0.71)	1.27** (1.08-1.49)
**Age**			
Age 20-34	2.48*** (1.83-3.37)	0.44*** (0.31-0.63)	0.88 (0.61-1.28)
Age 35-49	1.94*** (1.46-2.57)	0.62*** (0.45-0.85)	1.11 (0.79-1.56)
Age 50-64	1.52** (1.13-2.04)	0.86** (0.62-1.19)	1.00 (0.70-1.42)
Age 65 and over	Reference	Reference	Reference
**Marital status**			
Single	Reference	Reference	Reference
Married/common Law	0.94 (0.78-1.14)	1.26* (0.98-1.60)	1.05 (0.85-1.31)
Separated/divorced/widowed	0.90 (0.68-1.18)	1.60** (1.16-2.21)	0.89 (0.65-1.22)
**Education**			
Less than high school	Reference	Reference	Reference
High school	1.04 (0.74-1.46)	0.85 (0.58-1.26)	1.51* (0.96-2.36)
College or trades	1.14 (0.84-1.54)	0.73* (0.52-1.03)	2.02*** (1.35-3.03)
University	1.11 (0.81-1.51)	0.86 (0.60-1.22)	1.69** (1.12-2.55)
**Household income**			
Under $20,000	Reference	Reference	Reference
$20,000 to $49,999	1.52** (1.10-2.12)	0.88 (0.60-1.29)	1.02 (0.70-1.49)
$50,000 to $79,999	2.03*** (1.46-2.80)	0.81 (0.56-1.18)	0.83 (0.57-1.21)
$80,000 and over	2.65*** (1.92-3.65)	0.64**(0.44-0.94)	0.77 (0.53-1.11)
**Chronic condition**			
Yes	0.99 (0.83-1.18)	1.19(0.94-1.50)	1.01 (0.83-1.24)
No	Reference	Reference	Reference
**Self-perceived health**			
Excellent/very good	Reference	Reference	Reference
Good	0.87* (0.75-1.01)	1.13 (0.94-1.36)	1.12 (0.94-1.33)
Fair/poor	0.55*** (0.44-0.69)	1.35** (1.05-1.73)	1.44** (1.13-1.85)
			
**Self-perceived unmet health care needs**			
Yes	0.79** (0.67-0.93)	1.56*** (1.29-1.89)	1.28** (1.07-1.54)
No	Reference	Reference	Reference
**Rural residence**			
Urban	Reference	Reference	Reference
Rural	0.92 (0.75-1.13)	0.99 (0.77-1.28)	1.30** (1.04-1.63)
**Place of residence**			
CMA/CA	Reference	Reference	Reference
Strongly Influenced	1.20 (0.88-1.64)	0.73 (0.49-1.08)	0.94 (0.66-1.11)
Moderately Influenced	0.84 (0.59-1.20)	0.69 (0.43-1.11)	1.25 (0.84-1.85)
Weakly/Not Influenced	1.17 (0.75-1.82)	0.38**(0.19-0.76)	1.08 (0.66-1.75)
			
Constant	0.450	0.468	0.115
Observations	4,328	4,328	4,328
Pseudo R2	0.079	0.068	0.027

^a ^Notes: The dependent variable is consulting an alternative health care provider. The model used for estimation is logistic regression. 95% confidence intervals in parentheses. (*) significant at 10%, (**) significant at 5%, (***) significant at 1%. Reference categories are included in the table.

The results for acupuncture consultations are shown in Model 3. Some care must be taken when interpreting the significant odds ratios. Unlike in Models 1 and 2, women (OR = 0.60) and each of the three younger age groups (age 20 to 34, OR = 0.44; age 35 to 49, OR = 0.62; age 50 to 64, OR = 0.86) were less likely to seek care from an acupuncturist. These results do not mean that men and seniors are more frequent users of acupuncture. As the descriptive statistics in Table 2 attest to, these findings suggest that when men and seniors seek alternative health care, they are more likely to consult an acupuncturist than any other type of alternative health care provider. Model 3 also indicates that married/common law people (OR = 1.26) and those that are separated/divorced/widowed (OR = 1.60) were more likely to see an acupuncturist while those with a college or trades certificate (OR = 0.73) and people with a household income of $80,000 or more (OR = 0.64) were less likely to see this type of provider. Those reporting 'fair/poor' health (OR = 1.35) and people who said they had unmet health care needs (OR = 1.56) were more likely to consult an acupuncturist.

Finally, Model 4 displays the results for consultation with a homeopath or naturopath provider. Women (OR = 1.27), people with a college or trades (OR = 2.20) and university (OR = 2.20) education as well as those reporting 'fair/poor' health (OR = 1.44) and unmet health care needs (OR = 1.28) were all more likely to seek care from this type of provider. From a geographic perspective, rural residents (according to the Census rural definition) in Ontario were 1.3 times more likely (OR = 1.30) to consult a homeopath or naturopath provider.

### Three-Way Contingency Tables: Women and Health

The final step in the data analysis is to assess the relationship among three factors: women's health, unmet health care needs and alternative health care. The descriptive statistics and regression models showed that women were more likely than men to seek alternative health care. The analysis also revealed that the presence of a chronic condition and self-perceived unmet health care needs were important factors in influencing the use of alternative care. An analysis of the 2005 CCHS data indicates that women are more likely than men to suffer from certain chronic medical conditions including fibromyalgia, high blood pressure, chronic fatigue syndrome, and chemical sensitivities.

**Figure 2:**
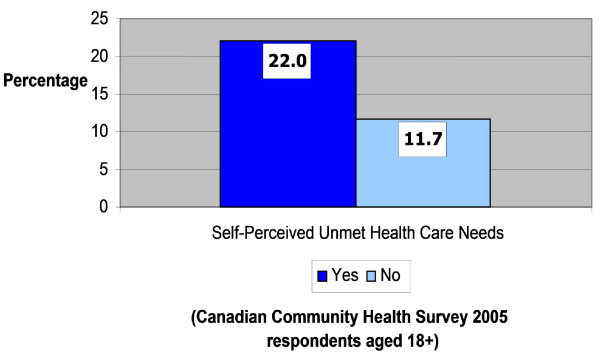
**Percentage of population consulting an alternative health care provider with self-perceived unmet health care needs**.

**Figure 3:**
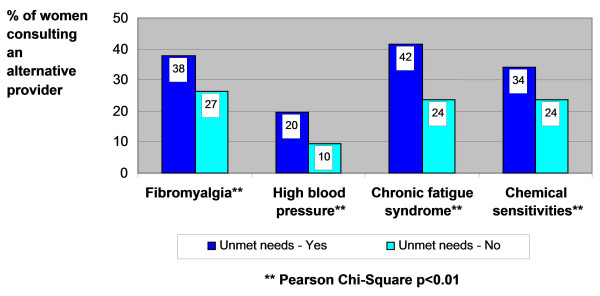
**Percentage of women reporting unmet health care needs consulting an alternative health care provider, by chronic condition**.

Figure 2 shows the relationship between alternative health care consultations (for both men and women in Ontario) and self-perceived unmet health care needs. It indicates that people who reported unmet health care needs (22%) were nearly twice as likely to see an alternative health care provider as those who said they had no unmet health needs (12%). This analysis is taken a step further in Figure 3, which displays the proportion of women suffering from one of four chronic medical conditions who consulted an alternative provider according to their perceptions of unmet health care needs. The data reveals a statistically significant association between this perception and alternative health care consultation. For instance, 38% of women in Ontario who have fibromyalgia and who felt they had unmet health care needs consulted an alternative provider compared to 27% of women with the same condition who reported no unmet health care needs. As shown in Figure 2, this trend is also evident for women with high blood pressure, chronic fatigue syndrome and chemical sensitivities. These results suggest that many women in Ontario feel that their health care needs are not being adequately met within the traditional medical system, particularly as related to the chronic conditions that affect women more than men.

## Discussion

The data analysis found that geography, with one notable exception, is not a factor in determining the use of alternative health care. While the total number of people using these services is much higher in urban areas, there is a fairly even proportional use across the urban to rural continuum and rural residents are just as likely to consult an alternative care provider as their urban counterparts. This suggests that the need for alternative care is just as high in the rural and remote areas of Ontario and that, from a business perspective, alternative care providers find it financially viable to set up in these areas, although greater travel distances are often a factor.

Interestingly, the analysis revealed that rural residents, as a whole, are more likely to consult a homeopath or naturopath provider than their urban counterparts, although there are no differences across the three rural MIZ. As discussed, relatively little is known about the use of alternative health care in the rural areas of Canada and other developed countries. Several U.S. studies have examined complementary and alternative medicine (CAM) in rural areas [28-30]. A study of Western North Carolina found that 'home remedies' is the most extensively used CAM among rural residents. Arcury et al suggest that greater use of 'home remedies' can be expected in rural areas as "rural residents are traditional in their attitudes and behaviors and because they have had less access to conventional care" [28]. However, the authors go on to say that while some CAM modalities such as chiropractic and homeopathy have a history of use in rural communities, "an accurate appraisal of CAM use in rural areas is needed" [28]. The findings, with respect to the use of homeopathy and naturopathy in rural Ontario, call for additional research. One possible avenue is a qualitative study involving interviews with practitioners and users in rural and remote regions of Ontario. Such a study could examine the possibility that alternative health care providers are somehow substituting for a lack of medical services in these areas, which are recognized as being underserved [22,24,31]. At the same time, the social and cultural aspects related to the popularity of homeopathy and naturopathy in rural communities could be explored.

The study demonstrated that there is a strong association between alternative health care use and certain socio-demographic characteristics including age, sex, education, health and self-perceived unmet health care needs. These demographic and health trends are largely consistent with the existing literature on alternative health care in North America, Europe and Australia. The association between higher socio-economic status and the use of alternative health care in Ontario could be related to the fact that most of these services are paid for out of pocket and individuals with higher levels of education may have better access to information through the Internet or their social networks and may be more inclined to acquire a broader knowledge of these alternative treatments.

Massage therapy is the most common type of alternative health care followed by homeopathy/naturopathy and acupuncture. The growing popularity of massage therapy can be attributed to the work of professional associations and organizations that have promoted the benefits of massage as preventative therapy, often used to manage stress and thereby sustain health [32]. A growing number of occupations requiring computer work have resulted in people spending long periods of time at a terminal leading to various repetitive strain injuries (such as carpo-tunnel syndrome), which can be relieved by way of massage therapy. However, in recent years, there has been growing concern in Ontario over the proliferation of unlicensed massage clinics especially in large cities [33] and the illegal misrepresentation of some providers as Registered Massage Therapists (RMT) [34]. The College of Massage Therapists of Ontario is concerned with these issues and strives to protect the public by upholding the standards and regulations associated with massage therapy in the province [34].

The paper found that health is strongly associated with alternative health care consultations - people with a chronic condition, lower health status, and self-perceived unmet health care needs are more likely to see an alternative health care provider, a situation supported by the literature [8,10,24]. The paper underscored the importance of women and health as related to alternative health care. The analysis found that women with chronic conditions such as fibromyalgia, high blood pressure, chronic fatigue syndrome and chemical sensitivities are more likely to see an alternative provider if they feel their health care needs are not being met. The treatment of these chronic conditions has had limited success by way of the traditional biomedical model. This certainly may be the reason why alternative health care practices are sought out for the treatment of these and other chronic conditions, often alongside conventional medicine [9]. In a study by Thorne et al, women with chronic illness revealed that their decision to use alternative health care reflected the fact that living with a complex chronic illness demands a dynamic approach to health care. They sought to gain knowledge about alternative modes of health care and take personal responsibility for their health as a way to improve their quality of life [35]. Alternative health care practices are known to be more holistic in nature and focus on environmental and social aspects of health, illness and wellbeing and often involve social and spiritual elements of care. For example, massage therapy involves an interpersonal intimacy between the patient and the practitioner [36]. Women are more likely to respond positively to the treatment of the 'whole person' and this could be a factor in explaining their higher use of alternative health care. Related to the issue of women and health, Adams et al's study of alternative health care use during pregnancy highlighted several gaps in the literature: the need for data on the lived-experience of alternative health care use in pregnancy, the lack of research examining patterns across culture over time, as well as the significance of the therapeutic encounter between practitioners and patients [37].

## Conclusion

The findings of this study support the recent literature on alternative health care as it relates to the socio-demographic and health characteristics of users. With the exception of the use of homeopathy/naturopathy in rural areas of Ontario, geography is not a factor. Much of the existing research is based on large household surveys (such as the CHHS) or administrative databases and there remains a need for more qualitative, place-specific research that explores the reasons why people use specific types of alternative health care as tied to socio-economic status, health, place of residence, and knowledge of these treatments.

## Competing interests

The authors declare that they have no competing interests.

## Authors' contributions

AW initiated the writing of the paper, defined the objectives and purpose and conducted the major revisions to earlier drafts as well as the final draft. PK conducted the data analysis and modeling based on the survey, contributed to the discussion section and completed the writing of the results section. JE conducted the literature review and completed the writing of both the introduction and the discussion. All authors read and approved the final manuscript.

## Pre-publication history

The pre-publication history for this paper can be accessed here:

http://www.biomedcentral.com/1472-6882/11/47/prepub

## References

[B1] Williams AM The diffusion of alternative health care: A Canadian case study of chiropractic and naturopathic health practices Canadian Geographer 2000 44 2 152 166 10.1111/j.1541-0064.2000.tb00699.x

[B2] Massage.ca Massage Therapy Glossary 2011 http://www.massage.ca/therapy_glossary.html Author Accessed March 4, 2011

[B3] Medicinenet.ca Acupuncture 2011 http://www.medicinenet.com/acupuncture/article.htm#1whatis Author Accessed March 4, 2011

[B4] Ontario Homeopathic Association 2010 http://www.ontariohomeopath.com Author Accessed March 4, 2011

[B5] Canadian Association of Naturopathic Doctors 2011 http://www.cand.ca Author Accessed March 4, 2011

[B6] Bishop FL Lwith GT Who Uses CAM? A Narrative Review of Demographic Characteristics and Health Factors Associated with CAM Use Evidence-Based Complementary Alternative Medicine 2010 7 1 11 28 10.1093/ecam/nen023 PMC2816378 18955327

[B7] de Bruyn T A Summary of National Data on Complementary and Alternative Health Care - Current Status and Future Development: A Discussion Paper 2002 Health Canada http://www.hc-sc.gc.ca/dhp-mps/pubs/complement/cahc-acps-summary-synthese/index-eng.php Accessed February 20, 2011

[B8] Park J Use of Alternative Health Care Health Reports 2005 16 2 39 42 Statistics Canada15971514

[B9] Wiles J Rosenberg MW 'Gentle caring experience': Seeking alternative health care in Canada Health and Place 2001 7 209 224 10.1016/S1353-8292(01)00011-9 11439256

[B10] Jones JF Maloney EM Boneva RS Jones A Reeves WC Complementary and alternative medical therapy utilization by people with chronic fatiguing illnesses in the United States BMC Complementary and Alternative Medicine 2007 7 12 10.1186/1472-6882-7-12 PMC1878505 17459162

[B11] Metcalfe A Williams J McChesney J Patten SB Jette N Use of complementary and alternative medicine by those with a chronic disease and the general population - results of a national population based survey BMC Complementary and Alternative Medicine 2010 10 58 10.1186/1472-6882-10-58 PMC2967501 20955609

[B12] MacLennan AH Myers SP Taylor AW The continuing use of complementary and alternative medicine in South Australia: costs and beliefs in 2004 MJA 2004 184 1 27 31 10.5694/j.1326-5377.2006.tb00092.x 16398628

[B13] Thomas K Coleman P Use of complementary or alternative medicine in a general population in Great Britain. Results from the National Omnibus survey Journal of Public Health 2004 26 2 152 157 10.1093/pubmed/fdh139 15284318

[B14] Xue CCL Zhang AL Lin V Da Costa C Story DF Complementary and Alternative Medicine Use in Australia: A National Population-Based Survey The Journal of Alternative and Complementary Medicine 2007 13 6 643 650 10.1089/acm.2006.6355 17718647

[B15] Hanssen B Grimsgaard S Launsø L Fønnebø V Falkenberg T Rasmussen NKR Use of complementary and alternative medicine in the Scandinavian countries Scandinavian Journal of Primary Health Care 2005 23 57 62 10.1080/02813430510018419 16025876

[B16] Fox P Coughlan B Butler M Kelleher C Complementary alternative medicine (CAM) use in Ireland: A secondary analysis of SLAN data Complementary Therapies in Medicine 2010 18 2 95 103 10.1016/j.ctim.2010.02.001 20430292

[B17] Wolsko P Ware L Kutner J Lin C Albertson G Cyran L Schilling L Anderson RJ Alternative/Complementary Medicine: Wider usage than generally appreciated The Journal of Alternative and Complementary Medicine 2000 6 4 321 326 10.1089/10755530050120682 10976978

[B18] Andrews GJ Boon H CAM in Canada: places, practices, research Complementary Therapies in Clinical Practice 2005 11 21 27 10.1016/j.ctcp.2004.10.004 15984220

[B19] Wu P Fuller C Liu X Lee H Fan B Hoven C Mandell D Wade C Kronenberg F Use of Complementary and Alternative Medicine Among Women With Depression: Results of a National Survey Psychiatric Services 2007 58 3 349 356 10.1176/appi.ps.58.3.349 17325108

[B20] Steinsbekk A Adams J Sibbritt D Jacobsen G Johnsen R The profiles of adults who consult alternative health practitioners and/or general practitioners Scandinavian Journal of Primary Health Care 2007 25 2 86 92 10.1080/02813430701267439 17497485 PMC3379753

[B21] Bodeker G Kronenberg F Burford G Bodeker G, Burford G Policy and public health perspectives on traditional, complementary and alternative medicine: an overview Traditional, complementary and alternative medicine: policy and public health perspectives 2007 London: Imperial College Press

[B22] Wardle J Lui C Adams J Complementary and alternative medicine in rural communities: current research and future directions The Journal of Rural Health 2010 (pre-publication view) 10.1111/j.1748-0361.2010.00348.x 22236320

[B23] Meyer S The spatial pattern of complementary and alternative medical offices across Ontario and within intermediate-sized metropolitan areas Urban Geography 2008 29 7 662 682 10.2747/0272-3638.29.7.662

[B24] Meyer S A geographic assessment of 'total' health care supply in Ontario: complementary and alternative medicine and conventional medicine The Canadian Geographer 2010 54 1 104 122 10.1111/j.1541-0064.2009.00285.x

[B25] Andrews GJ Wiles J Miller KL The geography of complementary medicine: perspectives and prospects Complementary Therapies in Nursing & Midwifery 2004 10 175 185 10.1016/j.ctnm.2004.05.003 15279859

[B26] Ramsay C Unnatural Regulation: Complementary and Alternative Medical Policy in Canada 2009 Vancouver, B.C: Fraser Institute

[B27] Vogel L Hodge-podge' regulation of alternative medicine in Canada Canadian Medical Association Journal 2010 182 12 10.1503/cmaj.109-3325 PMC2934827 20682736

[B28] Arcury TA Preisser JS Gesler WM Sherman JE Complementary and Alternative Medicine Use among Rural Residents in Western North Carolina Complementary Health Practice Review 2004 9 93 102

[B29] Gesler WB The Place of Chiropractors in Health Care Delivery: A Case Study of North Carolina Social Science and Medicine 1988 26 8 785 792 10.1016/0277-9536(88)90172-4 3287634

[B30] Shreffler-Grant J Hill W Weinert C Nichols E Ide B Complementary Therapy and Older Rural Women: Who Uses It and Who Does Not? Nursing Research 2007 55 1 28 33 10.1097/00006199-200701000-00004 17179871

[B31] Williams AM Restructuring home care in the 1990s: Geographical differentiation in Ontario, Canada Health & Place 2006 12 222 238 10.1016/j.healthplace.2004.09.002 16338637

[B32] Massage Therapy Canada http://www.massagetherapycanada.com Author accessed May 9, 2011

[B33] Kuitenbrouwer P Alcoba N Massage parlours proliferating in Toronto The Star Phoenix 2011

[B34] College of Massage Therapists of Ontario http://www.cmto.com Author accessed May 9, 2011

[B35] Thorne S Paterson B Russell C Schultz A Complementary/alternative medicine in chronic illness as informed self-care decision making International Journal of Nursing Studies 2002 39 671 683 10.1016/S0020-7489(02)00005-6 12231024

[B36] Moyer CA Rounds J Hannum JW A Meta-Analysis of Massage Therapy Research Psychological Bulletin 2004 130 1 3 18 14717648 10.1037/0033-2909.130.1.3

[B37] Wardle J Lui C Adams J Complementary and alternative medicine in rural communities: current research and future directions The Journal of Rural Health 2010 10.1111/j.1748-0361.2010.00348.x 22236320

